# Mismatch task conditions and error related ERPs

**DOI:** 10.1186/1744-9081-6-14

**Published:** 2010-02-23

**Authors:** Irene S Karanasiou, Charalabos Papageorgiou, Eleni I Tsianaka, Miltiades Kyprianou, George K Matsopoulos, Errikos M Ventouras, Nikolaos K Uzunoglu

**Affiliations:** 1 Institute of Communications and Computer Systems, National Technical University of Athens, 9, Iroon Polytechneiou str, 157 73 Zografou Campus, Athens, Greece; 2 School of Medicine, National and Kapodistrian University of Athens, Greece; 3 University Mental Health Research Institute (UMHRI), Athens, Greece; 4 Technological Education Institution of Athens, Greece

## Abstract

**Background:**

The N200 component of event related potentials (ERPs) is considered an index of monitoring error related responses. The aim of the present work was to study the effect of mismatch conditions on the subjects' responses in an auditory identification task and their relation to the N200 of stimulus-locked ERPs.

**Methods:**

An auditory identification task required to correctly map a horizontal slider onto an active frequency range by selecting a slider position that matched the stimulus tone in each trial. Fourteen healthy volunteers participated in the study and ERPs were recorded by 32 leads.

**Results:**

Results showed that the subjects' erroneous responses were equally distributed within trials, but were dependent on mismatch conditions, generated by large differences between the frequencies of the tones of consecutive trials. Erroneous trials showed a significantly greater negativity within the time window of 164-191 ms after stimulus, located mainly at the Cz and Fz electrodes. The LORETA solution showed that maximum activations, as well as maximum differences, were localized mainly at the frontal lobe.

**Conclusions:**

These findings suggest that the fronto-central N200 component, conceived an index of "reorientation of attention", represents a correlate of an error signal, being produced when representation of the actual response and the required response are compared. Furthermore the magnitude of the amplitude of the N200 rests on the relation between the present and the previous stimulus.

## Background

To adapt ongoing behavior in a changing world, human beings have to compare performed actions against their intended outcome; in this process the detection of errors is of major importance [1].

Interest in behavioral monitoring and evaluative processes has been heightened by the discovery of an event-related potential (ERP) component referred to as the error-related negativity (ERN) [2] or error negativity (Ne) [3]. It is important to note that in the performance -oriented cognitive literature there are reports indicating that not only the ERN/Ne waveforms but also the N200 component of ERPs might be conceived as an index of monitoring of errors.

The N200 is a frontocentral negative wave peaking between 100 and 250 ms after stimulus onset that is larger to negative than positive feedback, regardless of sensory modality of the feedback signal, and is also larger to negative than positive outcomes in psychophysiological tasks [4,5]. There is good evidence suggesting that anterior negativities in the N200 latency range are elicited by a variety of manipulations that tax error related response and which likely counts no-go N200 [6-8], feedback related negativity tasks [5,9], as well as N2 conflict tasks [10-12].

The mismatch negativity (MMN), formerly categorized as the early N2a subcomponent of the N200 [4,13,14], is a change-specific component of the event-related brain potential (ERP), initially observed in the auditory modality and later studied in the other sensory modalities too. The MMN is elicited when there is a change in the input, relative to the predictions formed on the basis of a memory trace of previous input. Within this framework the MMN would result from a failure to predict bottom-up input and consequently to suppress prediction error [15-17]. Recent work has linked the early component (in the range of about 100-140 ms) to a sensorial, or non-comparator account of the MMN, originated in the temporal cortex, and the later component (in the range of about 140-200 ms) to a comparator based mechanism of the MMN, involving the prefrontal cortex [18,19].

Despite the immense number of MMN studies, our understanding of the processes and mechanisms upon which the elicitation of MMN depends is rather incomplete. For a long time, it was debated whether representations of individual auditory features or an integrated representation of an event's combined features perform the automatic comparison. The MMN can be elicited by a variety of stimulus changes, ranging from simple changes in a single stimulus feature to abstract changes in the relationship between stimuli [20]. The MMN has been also observed in stimulus paradigms containing no frequently repeating sound [21].

Additionally, studies have analysed the mismatch negativity and error negativity as indices of error commission and monitoring. On error trials during a go/no-go auditory oddball task, the mismatch negativity amplitude was clearly reduced as compared with mismatch negativity amplitude on correct trials [22]. In two experiments where the perception of vowels belonging to two linguistically related languages was investigated, the results showed that the larger the acoustic difference, the larger the MMN amplitude. Acoustic difference between the stimulus pairs was reflected both by the MMN amplitude and reaction time speed (RTs). The MMN amplitude increased and the RTs decreased as the difference between the standard and deviant stimuli increased [23]. Short reaction times are related to increased error rates [24].

Recently, our team studied error related potentials in an auditory identification task. The aim for subjects participating in this task was to correctly map an active tone-frequency range onto a horizontal bar, by selecting a slider position that matches a stimulus tone that was presented to him/her in the beginning of each trial. Our team examined the patterns of brain activity of actors and observers elicited upon receiving feedback information of the actor's response and obtained findings suggesting that feedback information has a different effect on the intensities of the ERP patterns of actors and observers depending on whether the actor committed an error [25]. Part of the data obtained during this experiment in the single-participant conditions, that have not been previously reported, were processed in the framework of the present study focusing on stimulus locked potentials.

More specifically, our purpose was twofold. Firstly, our aim was to examine the patterns of stimulus-locked N200 waveforms during frequency mismatch detection in relation to correct versus incorrect responses. The frequency mismatch was studied between consecutive stimulus tones as well as stimulus and corresponding response-related feedback tones. Secondly, using the LORETA technique, our aim was to investigate candidate brain structures that are responsible for the N200 differences observed under mismatch conditions.

## Methods

### Participants

Fourteen healthy individuals (eight men and six women), with mean age of 26.6 ± 2.9 years and high level education (education years 17.7 ± 2.3), all with normal hearing as measured by pure-tone audiograms (thresholds <15dB HL), participated in the experiment. The male and female subgroups were homogeneous with regards to age and educational level. All the participants were right-handed and had no history of any hearing problem. Approval was obtained by the institutes' ethics committee and informed consent was obtained from all subjects.

### Stimuli and procedures

In the present research an auditory identification task has been used. The person who performed the task sat in front of a computer screen and at each trial he/she heard the stimulus tone (stimulus frequency) with duration of 1 sec presented through the headphones (K44 headphones, AKG).

The stimulus tone was randomly selected for each trial within a fixed frequency range (200-600 Hz) which remained the same throughout the whole experiment. An LCD monitor with refresh rate 60 Hz and a customized program for stimulus presentation were used.

The participant's task was to position a slider presented on the computer screen with a gamepad, such that the slider position would match the frequency of the stimulus tone. At the beginning of the trial blocks, the starting position of the cursor was in the middle of the slider and the participants did not know the scaling of the frequency range within which the slider position should be mapped. After the positioning of the slider, the frequency corresponding to the participant's selected slider position (response frequency) was presented to the participant. The experiment consisted of 40 trials for each participant.

Before the experiment, the subjects were submitted to an acoustic pre-test in order to examine their hearing ability in the frequency range that was used in the experiment. During this test two tones were presented to the participants. Then, the participants had to identify which of these tones was higher than the other. The frequencies of the two tones selected for the acoustic test were determined as the 25% and the 75% of the range of 400 Hz bandwidth. The subjects heard the tones with their headphones and responded orally to the experimenter. All participants were capable to discriminate between the tones presented in the pre-test.

### EEG recordings and experimental setup

The experimental setup included a Faraday room, which screened any electromagnetic interference that could affect the measurements. The EEG was recorded continuously using a 32-channel electrode cap (Biosemi, Active Two system) according to the international 10-20 EEG system [26]. The electrodes used were Fp1, Fp2, Pz, Fz, O1, O2, P3, P4, P7, P8, C3, C4, T7, T8, F3, F4, F7, F8, Cz, Oz, CP5, CP6, CP1, CP2, FC1, FC2, FC5, FC6, AF3, AF4, PO3 and PO4.

Galvanic isolation of the participants was ensured by using an optical receiver (Biosemi New USB2 Receiver) for trigger inputs, while in parallel, interference pickup was also eliminated. The electrode cables were also bundled to eliminate potential magnetic interference. The vertical electro-oculogram (EOG) was recorded bipolarly from electrodes placed above and below the eyes and the horizontal EOG was monitored from electrodes at the outer canthi of the eyes. The data were filtered off-line, high-pass at 0.05 Hz and low-pass at 35 Hz with a zero-phase digital filter in both forward and reverse directions.

All signals were digitized with a sampling rate of 256 Hz. All scalp signals were referenced online to both mastoids, but were later offline re-referenced to the average of all scalp electrodes. Trials were averaged to ERPs separately for each condition and each subject, relative to a 100 ms pre-stimulus baseline. To eliminate EOG artifacts, trials with EEG voltages exceeding 80 μV were rejected from the average.

### Categorization of correct and erroneous responses

The auditory frequency perception resolution in humans can be described in terms of an Equivalent Rectangular Bandwidth (ERB) around a stimulus frequency (Sf). According to psychoacoustics theory, the Equivalent Rectangular Bandwidth (*Be*) in Hz can be approximated according to the following formula: *B*_*e *_= 6.23 10^-6 ^*f*^2 ^+ 9.339 10^-2 ^*f *+ 28.52, where *f *is the frequency of the sound. The discrimination between the participant's erroneous and non-erroneous responses was performed with the use of the above formula. A subject's response was considered correct if the response frequency (Rf) was in the range between Sf-Be/2 and Sf+Be/2. For each trial starting from the second, two new variables were calculated. The first one was the absolute frequency difference between the present and previous Sf (fd1). The second one was the absolute frequency difference between the present Sf and the previous Rf (fd2). Consequently the number of trials taken into consideration was 39, since the first trial did not have a previous stimulus and response frequency.

### LORETA source localization method

The low resolution brain electromagnetic tomography (LORETA) differentiates between structural and energetic processes related to information processing as revealed by the associated EEG/ERP waveform [27,28]. The structural level, revealed by the location of the local maxima of the current source density distribution, describes the time dependent network of activated brain areas. The magnitude of the source strength, a measure of the energetic component, describes the allocation of processing resources [29]. The utilized LORETA version was registered to the Talairach brain atlas [30,31]. The solution space consisted of 2394 voxels with a spatial resolution of 7 mm. LORETA images were constructed for each subject averaging for his erroneous and correct responses, followed by voxel-by-voxel pairwise t-test comparisons. The structure probability maps atlas [31] was used to identify which brain regions were involved in the ERP waveforms as well as in differences between the compared conditions (error vs correct responses). Brain regions corresponding to the observed locations identified by the Talairach coordinates are reported [30,31].

### Statistical analysis

The overall ratio of erroneous/correct responses was 275/271. This ensured for all subjects an adequate signal to noise optimization. Differences of fd1 and fd2 between the correct and erroneous responses were calculated using the paired t-test.

All ERP analyses were performed using the LORETA software. Specifically the input data were the average ERP values for each subject separately for the correct and error condition at each time frame from -100 to 500 ms around the stimulus tone and at each of the 32 electrodes. Thus for each subject and each condition (i.e. correct and erroneous response) the input data comprised 180 time frames × 32 electrodes giving a total of 5760 averaged ERP values.

These initial data were subjected to paired samples topographic analysis of variance (TANOVA), as well as paired samples electrode-wise comparison of ERPs. The t-values were calculated via randomization using a Monte-Carlo method and were corrected for multiple comparisons [32]. The purpose of this analysis was twofold. Firstly, to establish the time windows where differences between the two conditions are maximal and achieve statistical significance. Secondly, to locate the electrodes that were most instrumental in the formation of these differences.

Subsequently the ERP data were converted to LORETA files using the inverse solution of the software [33]. This procedure yields the brain activation maps at 2394 voxels. In the same manner these LORETA files were subjected to paired samples voxel-wise comparisons at each time frame separately. Once again, the purpose was to find the time window where statistically significant differences between the two conditions are maximal, both in terms of absolute differences, and in terms of the span of the time window itself. Finally, the application of pairwise comparisons of the average activation maps within the specific window revealed the most consequential clusters of voxels that differentiate the two conditions. The level of statistical significance throughout the tests was set at 0.05.

This functional mapping of the human brain provides a means to identify both the temporal and spatial characteristics of the differences between the two conditions, providing clues to the underlying mechanisms that qualify these differences.

## Results

Preliminary analysis revealed that the subjects' erroneous responses within the 39 trials were identically distributed. Conversely, the paired t-test revealed statistical significant differences of the mean values of fd1 between the correct (138 ± 96 Hz) and erroneous (161 ± 89 Hz) responses (mean difference = 23 Hz, t(544) = 2.96, p = 0.003). Even greater are the differences of the mean values of fd2 between the correct (125 ± 99 Hz) and erroneous (154 ± 89 Hz) responses (mean difference = 29 Hz, t(544) = 3.60, p < 0.001).

**Figure 1:**
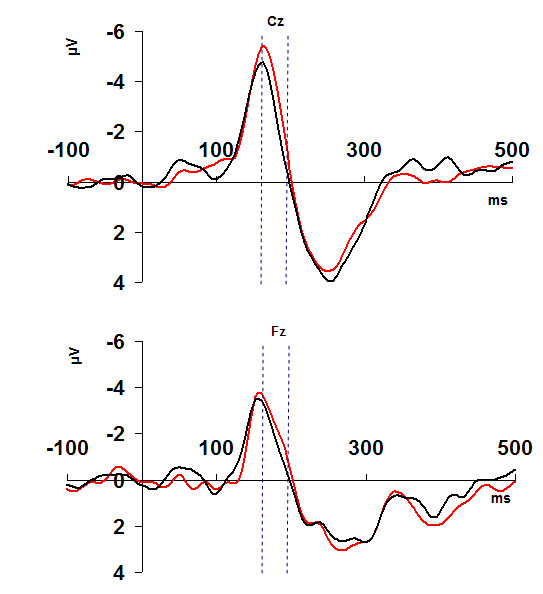
**Mean amplitude values at Cz and Fz electrodes around the stimulus tone**. Amplitude values of the Cz and Fz electrodes in the time window -100 to 500 ms around the stimulus tone depending on the subjects' subsequent correct and erroneous responses. Black lines depict the correct responses, red lines depict the errors. Dotted blue lines mark the time window of statistical significant differences between the two conditions

The TANOVA procedure revealed a time window at 164-191 ms where differences between the two conditions achieved statistical significance (p < 0.05). Subsequent paired samples electrode-wise comparisons of the ERPs showed that electrodes Cz and Fz are the most consequential in the significant between conditions differences within this time window. As Figure 1 shows, both electrodes exhibit within this time window a significant negative peak.

**Figure 2:**
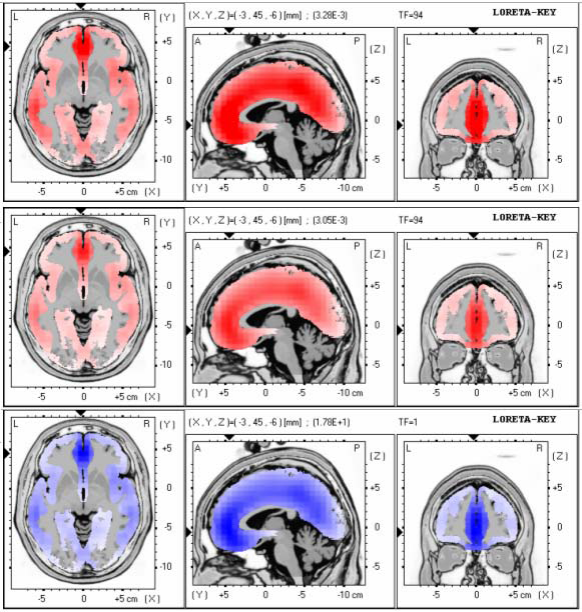
**Activation maps for the correct and error responses of the subjects for the time window 164-191 ms**. Activation maps for the error (top map) and correct (middle map) responses of the subjects for the time window 164-191 ms. Differences (in t-values) between the activation maps for the two conditions (bottom map).

Figure 2 shows the LORETA solution at this negative peak for the two conditions. Maximum activation for both conditions was localized at (X = -3, Y = 45, Z = -6) having best matches in the Talairach atlas at Brodmann areas 10 and 11, medial frontal gyrus, frontal lobe and at Brodmann area 32, anterior cingulate, limbic lobe. The more intense red colour for the erroneous responses in contrast to correct responses indicates greater activation of the specific regions. Interestingly the voxel-wise comparison of the activation maps between the two conditions was localized at the same area (X = -3, Y = 45, Z = -6, t-value = 3.55, p < 0.05). This means that maximal differences between the two conditions occur at the areas of maximum activation.

## Discussion

In the present research we examined the patterns of stimulus-locked N200 waveforms corresponding to subsequent correct versus incorrect responses in relation to the frequency deviation between consecutive stimulus tones and the frequency deviation between the present stimulus tone and response-related feedback tone that the subject heard just before. Results showed that mismatch conditions between two consecutive trials increase the probability of errors, which are reflected in larger negative peaks of the N200 component at the fronto-central electrodes.

More specifically, greater values of fd1 that signify larger stimulus frequency differences between two consecutive trials create increased mismatch conditions for the subject to correctly identify the present stimulus frequency. The effect of mismatch on the subject's response is even more obvious with respect to fd2, which is the difference between the present frequency stimulus and the previous response frequency. For each stimulus the subjects responds by positioning the slider. Subsequently, the subject hears a tone corresponding to this position. The subject expects that, by means of simple comparison, this feedback information will aid him in the next trial. However, if the stimulus frequency of the next trial largely deviates from this response tone the value of this feedback information is essentially invalidated, resulting in greater probability of committing an error. Consequently, any stimulus that is not similar to the previous tone will act as an oddball, creating a mismatch which results in an increased N200 and leads also to an error. In other words, the appearance of stimulus locked N200 and of subsequent response errors both seem to originate from a common cause, namely the mismatch between the previous response and present stimulus frequencies. Stimulus frequencies that are close together promote the subject's ability to fine-tune his/her response. Conversely two consecutive frequencies that are further apart hinder the ears' and ultimately the brain's pattern-matching capabilities, which are reflected in the increased negative amplitudes of the stimulus-locked N200 component, and to inferior judgments.

The present results appear to be compatible with the concept of the N200 system, according to which N200 is based on a comparison between the current sound and a model-based concrete prediction of a forthcoming sound. An N200 auditory component, is commonly thought to reflect the outcome of a comparison process between the representation of the current event and a representation (memory trace, neural model) of the regularities in the event history. Once a mismatch between the two representations is detected, N200 is elicited [34-37]. This scheme appears to be in accordance with the model that N2 is related to the modulation of the early stages of response preparation and selection [38-40].

Moreover, the scalp distribution and the obtained neural generators of the N200 appear to be in line with the N2a source localization studies. The scalp-recorded MMN has its largest amplitude over the fronto-central scalp areas. Maximal differences between the two conditions also occur at the areas of maximum activation.

Numerous studies have consistently reported evidence for two main locations concerning the generators of the MMN. The one location is referred to the temporal cortex and the other to the frontal cortex. As mentioned in the introduction section, reported findings are consistent with the observation of two sub-components of the MMN; the early component (in the range of about 100-140 ms) and the later component (in the range of about 140-200 ms) [18]. The sources in the temporal areas are thought to be involved in processing changes of the sound's physical properties, whereas the sources on the frontal areas have been considered to reflect reorientation of attention [18,41].

A frontal-lobe involvement in MMN generation was already proposed on the basis of only four-channel scalp potential recordings [4,42,43]. This suggestion [14] was supported by later analyses of the MMN scalp-potential distribution, which indicated an MMN source in the frontal lobes [44,45]. Frontal MMN sources were also suggested by studies using source-current modelling [46] techniques. Furthermore, frontal MMN sources were also supported by intracranial ERP [47-49], PET [50], and fMRI recordings [41,51-55] as well as by developmental data [56]. The role of prefrontal also generators is supported by studies of patients with prefrontal lesions who showed diminished temporal MMN amplitudes [57].

Besides the frontal neural generators also the ACC (Anterior Cingulate Cortex) seems to be involved in the observed ERP results. The ACC is typically linked to errors [58,59]. The implication of the ACC in the present findings may be explained by the fact that the ERP comparisons are based on correct and erroneous responses.

## Conclusions

Analyses revealed that that there are significant differences in ERPs elicited at the stimulus tone depending on whether the subject's subsequent response was correct or erroneous. Both the differences in ERP patterns at the stimulus tone and the differences in the responses may be attributed to a common cause, which is the magnitude of the difference between the previous and the present stimulus tone as well as the previous response tone. It seems that a mismatch between two consecutive tones acts as an oddball, increasing the probability of the appearance of stimulus-locked N200 and a subsequent erroneous response. Finally, LORETA findings indicate that maximum activations, as well as maximal differences, occur mainly at the frontal lobe.

## Competing interests

The authors declare that they have no competing interests.

## Authors' contributions

ISK and EIT performed the acquisition of the EEG data. MK, ISK and EIT contributed to the statistical analysis of the data. CP, ISK and EIT participated to the interpretation of the results and composed the manuscript. GKM and EMV participated to the interpretation of the results. NKU conceived the core of the study design. NKU, GKM and EMV also revised the manuscript critically. All authors read and approved the final manuscript.
